# Population Pharmacokinetics of High-Dose Methotrexate in Chinese Pediatric Patients With Acute Lymphoblastic Leukemia

**DOI:** 10.3389/fphar.2021.701452

**Published:** 2021-07-13

**Authors:** Xuan Gao, Xiao-Wen Qian, Xiao-Hua Zhu, Yi Yu, Hui Miao, Jian-Hua Meng, Jun-Ye Jiang, Hong-Sheng Wang, Xiao-Wen Zhai

**Affiliations:** ^1^ Outpatient and Emergency Management Office, National Children’s Medical Center, Children’s Hospital of Fudan University, Shanghai, China; ^2^ Department of Hematology and Oncology, National Children’s Medical Center, Children’s Hospital of Fudan University, Shanghai, China

**Keywords:** methotrexate, population pharmacokinetics, NONMEM, acute lymphoblastic leukemia, pediatric patients

## Abstract

High-dose methotrexate (HD-MTX) is widely used in pediatric acute lymphoblastic leukemia (ALL) treatment regimens. In this study, we aimed to develop a population pharmacokinetic (PK) model of HD-MTX in Chinese pediatric patients with ALL for designing personalized dosage regimens. In total, 4,517 MTX serum concentration data for 311 pediatric patients with ALL, aged 0.75–15.2 years and under HD-MTX treatment, were retrospectively collected at a tertiary Children’s Hospital in China. The non-linear mixed-effect model was used to establish the population PK model, using NONMEM software. The potential covariate effects of age, body weight, and biochemical measurements (renal and liver function) on MTX PK disposition were investigated. The model was then evaluated using goodness-of-fit, visual predictive check. MTX PK disposition was described using a three-compartment model reasonable well. Body weight, implemented as a fixed allometric function on all clearance and volume of distribution parameters, showed a substantial improvement in model fit. The final population model demonstrated that the MTX clearance estimate in a typical child with body weight of 19 kg was 6.9 L/h and the central distribution of volume estimate was 20.7 L. The serum creatinine significantly affected the MTX clearance, with a 0.97% decrease in clearance per 1 μmol/L of serum creatinine. Other covariates (e.g., age, sex, bilirubin, albumin, aspartate transaminase, concomitant medication) did not significantly affect PK properties of MTX. The proposed population PK model could describe the MTX concentration data in Chinese pediatric patients with ALL. This population PK model combined with a maximum a *posteriori Bayesian* approach could be used to estimate individual PK parameters, and optimize personalized MTX therapy in target patients, thus aiming to reduce toxicity and improve treatment outcomes.

## Introduction

Acute lymphoblastic leukemia (ALL) is the most common pediatric hematological cancer, accounting for 26–30% of all cancers diagnosed in children up to 14 years of age and contributing to approximately 80% of all childhood leukemia cases (Desantis et al., 2014; Pavlovic et al., 2019). Methotrexate (MTX) is a folate analog widely used as the first-line chemotherapy in high-dose (HD) consolidation and low-dose maintenance therapy for childhood ALL. The antileukemic effect of MTX is attributed to its competitive inhibition of dihydrofolate reductase (Galivan, 1980; Schmiegelow, 2009). HD-MTX is defined as a dose higher than 500 mg/m^2^, which could elicit a broad range of antitumor activities (Evans et al., 1986; Howard et al., 2016; Kawakatsu et al., 2019; Shi et al., 2020). It has been reported that HD-MTX can reduce the relapse rate, increase the event-free survival rate, and suppress the development of central nervous system (CNS) leukemia in childhood ALL (Balis et al., 1985; Evans et al., 1986).

However, approximately 75% of the pediatric patients with ALL experienced therapy-related adverse effects (Gervasini and Vagace, 2012; Kawakatsu et al., 2019; Panetta et al., 2020), among them, 1–2% of the patients died from chemotherapy-associated toxicities (Hunger and Mullighan, 2015). Although the patients were provided aggressive folate supplementation, intravenous fluid hydration, and urine alkalinization during the course of HD-MTX therapy, the renal toxicity (i.e., renal dysfunction), at an approximate frequency of 1.8%, was observed often (Widemann et al., 2004). Renal toxicity leads to impaired MTX clearance and prolonged exposure to toxic concentrations, which can further deteriorate renal function and cause non-renal adverse events (AEs). Exposure to HD-MTX is highly associated with toxicity, including neurotoxicity (Bhojwani et al., 2014), hepatotoxicity (Cheng, 2008; Hegyi et al., 2012), mucositis (Cheng, 2008; Johansson et al., 2011), myelosuppression (Comandone et al., 2005; Joerger et al., 2010), and nephrotoxicity (Comandone et al., 2005; Yarlagadda and Perazella, 2008; Howard et al., 2016). These AEs often result in interruption or discontinuation of chemotherapy and increase relapse risks (Schmiegelow et al., 1989; Schmiegelow and Pulczynska, 1990; Schmiegelow, 2009).

MTX is primarily eliminated through the kidneys (up to 90% of the intravenous dose within 24 h) (Bleyer, 1978). It is metabolized to 7-hydroxy-MTX in the liver, which contributes to MTX activity (Csordas et al., 2013), and a small part of MTX is excreted in the bile with partial intestinal reabsorption. Several efflux and uptake transporters (e.g., BCRP, MRP2, MRP3, MRP4, OAT1, and OAT3) are involved in the pharmacokinetic (PK) disposition process of MTX, which could lead to substantial variability in PK exposure (Treviño et al., 2009; Leveque et al., 2011; Ramsey et al., 2013; Schulte et al., 2021). Recent studies suggested that MTX elimination varied significantly between HD-MTX courses, and extremely delayed MTX elimination was observed in approximately 0.5% of pediatric patients with ALL (Svahn et al., 2017). The routine therapeutic drug monitoring for MTX has been strongly recommended to reduce the incidence of AEs in the target patient population, as suggested by several clinical guidelines and drug labeling.

In general, conventional PK studies require the collection of a series of PK blood samples to compute the PK exposure parameters (i.e., Cmax, AUC) using a non-compartmental analysis approach. However, this approach is inapplicable for routine clinical therapeutic drug monitoring, especially in the pediatric population, since only sparse PK concentrations is available. The population PK approach has advantages such as unitizing sparse PK data in addition to quantifying and analyzing the covariate effect, and therefore is widely used in individual therapy. Although a number of population PK models of MTX have been established previously in various clinical settings, the extrapolation of the previous population PK models to a new clinical setting remains highly uncertain (as shown in the cases of vancomycin and ciclosporin) (Deng et al., 2013; Mao et al., 2018). Additionally, previous PK studies on MTX have suggested that more population PK studies are still needed due to the variation in patient demographic data and dosage regimens.

Considering these factors, the main objective of this study is to develop a population PK model of HD-MTX infusion in Chinese pediatric patients with ALL and to explore the potential effect of covariates that could affect MTX PK profiles.

## Materials and Methods

### Study Population

The study protocol and collection of retrospective clinical data were approved by the Research Ethics Committee of Children’s Hospital of Fudan University [No (2020) 444]. The study population identified was Chinese pediatric patients (≤ 18 years old) diagnosed with ALL who received HD-MTX at the Children’s Hospital of Fudan University, Shanghai, from January 2014 to December 2019.

### ALL Treatment Regimen

The pediatric patients with ALL were diagnosed and treated in accordance to the guidelines outlined in the China Children’s Leukemia Group (CCLG) ALL-2008 protocol (Cui et al., 2018) and China Children’s Cancer Group (CCCG) ALL-2015 protocol (Zhu et al., 2020). Children diagnosed with ALL were classified into the low-risk (LR), intermediate-risk (IR), and high-risk (HR) groups. MTX was administered as intravenous (IV) infusion over 24 h at a dosage regimen of 3 g/m^2^ in the LR group and 5 g/m^2^ for both IR and HR groups, respectively. Ten percent of the total dose was administered as a loading dose (0.5 h), followed by infusion of the remaining 90% of the dose over 23.5 h. In addition, after 1–2 h of starting MTX infusion, 6–12.5 mg dose (according to the patients’ age) of MTX was administered via intrathecal injection to prevent CNS ALL. The patients were also orally administered 6-mercaptopurine 25 mg/m^2^ daily for 14 days. The MTX dose for pediatric patients was reduced to a lower level in case of substantial delay in elimination in previous MTX chemotherapy. Biochemical tests (e.g., liver and renal function) were routinely conducted before (baseline), 48 h after, and at the end of MTX chemotherapy. According to clinical practice, findings of the renal and liver function tests for pediatric patients must be roughly normal (e.g., SCr) before MTX chemotherapy.

### Pharmacokinetic Sampling Collection

Routine samples for MTX concentration measurement for therapeutic drug monitoring were collected from the patients according to the clinical practice in hospital. As per clinical guidelines, delayed serum excretion was defined as an MTX concentration greater than 1.0 μmol/L at 42 h after the initiation of MTX infusion. Standard leucovorin rescue was administered at 15 mg/m^2^/dose (intravenous) starting at 42 h and administered every 6 h, and was modified according to MTX serum concentrations until the MTX concentration at 48 h was less than 0.25 μmol/L. If MTX serum concentrations were higher than 0.4 μmol/L, leucovorin rescue was continued every 6 h until the MTX concentration was less than 0.25 μmol/L. Therefore, the PK blood samples were routinely collected at 24, 42, and 48 h after the treatment, and additional PK samples were collected every 24 h afterward if clinically indicated. The majority of patients received multiple cycles of HD-MTX therapy and multiple MTX concentrations were measured in each cycle.

Demographic data, including age at treatment, sex, body weight, body surface area (BSA), body mass index, time-varying laboratory tests, including data on serum creatinine levels (SCr), alanine transaminase (ALT), aspartate transaminase (AST), alkaline phosphatase (ALP), total bilirubin (TBIL), direct bilirubin, and albumin, were collected. PK data, including the date and time of dosage administration and PK collection, as well as MTX concentrations were collected and appended to the demographic data of each patient. MTX serum concentrations were measured by fluorescence polarization immunoassay. The concomitant medication during the MTX chemotherapy was recorded in the medical record of the hospital’s HIS system. Medications known to alter MTX PK behaviors (e.g., dasatinib, imatinib, penicillin, omeprazole, and non-steroidal anti-inflammatory drug (NSAIDs)) during the MTX chemotherapy were selected for covariate analysis.

### Population Pharmacokinetic Analysis

A total of 311 children were enrolled in this population PK modeling analysis. A total of 4,517 blood samples were collected for MTX concentration measurements. Population PK analysis was performed using non-linear mixed-effects model in the NONMEM^®^ software (version 7.4, ICON Development Solutions, Ellicott City, MD, United States) and data were compiled using gFortran (version 4.60). Perl-speaks-NONMEM (PsN; version 4.6.0) and R language (version 3.4.0, http://www.r-project.org/) were used to visualize the outputs. The first-order conditional estimation algorithm with η-ε interaction (FOCE-I) was used throughout the model-building procedure. Discrimination between models during the model-building process was based on standard visual diagnostics and the objective function value (OFV), which were calculated to be proportional to twice the log-likelihood (−2LL). A decrease in OFV (∆OFV) of 6.64 was considered a significant improvement of model fit (*p* < 0.01) between the two hierarchical models after inclusion of one additional parameter (one degree of freedom difference).

MTX concentrations were logarithmically converted. A base model without incorporating any covariates and capable of describing the data appropriately was selected. During this step of analysis, all possible structural compartments were investigated, i.e., one-, two-, and three-compartment disposition models.

Inter-individual variability (IIV) was added exponentially to all PK parameters (Eq. 1).

$$\theta_{i}=\;\theta \cdot \text{exp}\left(\eta_{i,\theta }\right)$$ (1)

where $\theta_{i}$ is the individual parameter estimate for the *i*th individual, θ is the population estimate of the investigated PK parameter, and $\eta_{i,\theta }$ is the IIV of the investigated PK parameter, assumed to be normally distributed with a zero mean and variance ω^2^. The residual unexplained variability, assumed to be normally distributed with a zero mean and variance σ^2^, was modeled with an additive error on the natural log-transformed concentrations, which was approximately equivalent to an exponential residual error on an arithmetic scale.

### Covariate Modeling

Demographic data on body weight were added in the model as a simultaneous incorporation of an allometric function on all clearance and distribution volume parameters (Eq 2, 3, respectively).

$$CL_{i}=\;CL_{typical}\cdot {\left(\frac{BW_{i}}{BW_{median}}\right)}^{0.75}\cdot \exp\left(\eta_{i,CL}\right),$$ (2)

$$V_{i}=\;V_{typical}\cdot \left(\frac{BW_{i}}{BW_{median}}\right)\cdot \exp\left(\eta_{i,V}\right),$$ (3)

where BW_i_ is the individual body weight and BW_median_ is the median body weight of the patients (i.e., 19 kg) in this study *CL*_i_ and *V*_i_ are the individually predicted clearance and distribution of volume, respectively. *CL*_typical_ and *V*_typical_ are the typical clearance and distribution of volume value of the population, respectively.

The effect of age-related maturation on clearance was then evaluated using a saturation-type E_max_ function (Eq. 4).

$$CL_{i}=CL_{typical}\cdot \frac{Age}{Age_{50}+Age}\cdot {\left(\frac{BW_{i}}{BW_{median}}\right)}^{0.75}\cdot \exp\left(\eta_{i,CL}\right),$$ (4)

where $Age_{50}$ is the age associated with reaching 50% of the clearance maturation.

Other covariates (e.g., sex, liver function, renal function, and concomitant drugs) were investigated for all model parameters using a forward selection (*p* = 0.01) and backward elimination (*p* = 0.001) procedure. The concomitant drugs used in ≥5% of patients were evaluated for covariate effects.

In addition to statistical significance, the explanation of the variability by inclusion of a covariate was considered. A covariate was excluded from the model if the reduction in variability was <5%.

### Model Evaluation

Basic goodness-of-fit diagnostic plots were used to evaluate systematic errors and model misspecifications. The sampling importance resampling (SIR) was used to calculate parameter uncertainty in the final population PK model (samples = 2,000, resamples = 1,000). The overall predictive performance of the final model was evaluated using simulation-based diagnostics (i.e., using prediction-corrected visual predictive checks (Bergstrand et al., 2011), *n* = 1,000 simulations).

## Results

The PK data of 311 pediatric patients were included in the population PK modeling analysis. All patients received a total of 1,250 cycles of MTX chemotherapy. Demographic characteristics of the patients are shown in Table 1.

**TABLE 1:** **|** Demographic characteristics of 311 pediatric patients with ALL.

Characteristics	Median (range)
*Demographics*	
Male (*n*, %)	197 (63.3)
Age, years	5.0 (0.75–15.2)
Height, cm	112 (67–175)
Body weight, kg	19.0 (4.5–113.0)
Biochemical test	
Albumin, g/L	43.4 (23.8–56.5)
Total protein, g/L	63.4 (17.4–78.6)
Total bilirubin, µmol/L	5.9 (1.5–114.0)
Direct bilirubin, µmol/L	1.9 (0.1–45.0)
AST, U/L	22.6 (7.0–319.0)
ALT, U/L	16.0 (2.0–390)
Serum creatinine, µmol/L	26.0 (8.0–135.0)
MTX dosage regimen [n (course)]	
5 g/m^2^	142 (464)
4 g/m^2^	47 (84)
3 g/m^2^	154 (524)
2 g/m^2^	42 (107)
1 g/m^2^	28 (71)
Concomitant medication [*n* (course)]	
Dasatinib	13 (48)
Imatinib	4 (14)
Omeprazole	59 (142)
Sulphonamides	14 (15)
NSAIDs	24 (25)
Penicillin	6 (6)

Notes: The demographic data were summarized from 1,250 cycles of MTX chemotherapy. ALT: alanine transaminase, AST: aspartate transaminase.

An OFV of 7,229.251 was yielded when one disposition compartment model was used to fit MTX concentration-time data. By employing a two-compartment model, the model fit was improved significantly (∆OFV = −5,912.277), and the three-compartment model further improved the model fit substantially (∆OFV = −274.754).

Body weight, implemented as a fixed allometric function on all clearance and volume of distribution parameters, showed a substantial improvement in model fit (∆OFV = −268.666). Inclusion of age-related maturation effect on CL did not show a significant improvement in model fit further.

Inclusion of SCr in a linear function on clearance significantly improved model fit (∆OFV = −284.008), with slope estimates of −0.0097, and reduced the variability of Q_1_ by 29.2% (CV% of IIV from 37.7 to 26.7%). Adding TBIL on clearance (∆OFV = −30.975) and albumin in the central volume of distribution (∆OFV = −36.722) using linear function improved model fit significantly, with slope estimates of −0.0044 and −0.070, respectively. However, these two covariates did not substantially reduce either the inter-individual or residual variability further (i.e., <5%), as defined above and, therefore, were not included in the model. Other covariates (e.g., sex, AST, and ALT) did not significantly affect MTX PK properties.

The covariate effects of omeprazole (co-medicated in 19.0% of patients and 11.4% of total chemotherapy courses) and NSAIDs concomitant medication (co-medicated in 7.7% of patients and 2.0% of total chemotherapy courses) were investigated. Their inclusion on clearance improved model fit significantly (∆OFV = −64.331 and −42.874, respectively); however, the reduction in either inter-individual or residual variability was minimal (<1.1%). Therefore, these two co-medications were not retained in the final model. Only 13 (4.2%) and 4 (1.3%) pediatric patients with BCR-ABL1-positivity received treatment with dasatinib and imatinib, respectively, and six pediatric patients received penicillin, which are known to delay MTX elimination. However, the effect of this concomitant therapy was not investigated in the covariate analysis due to the small number of patients (<4.2%) receiving these medications.

**FIGURE 1:**
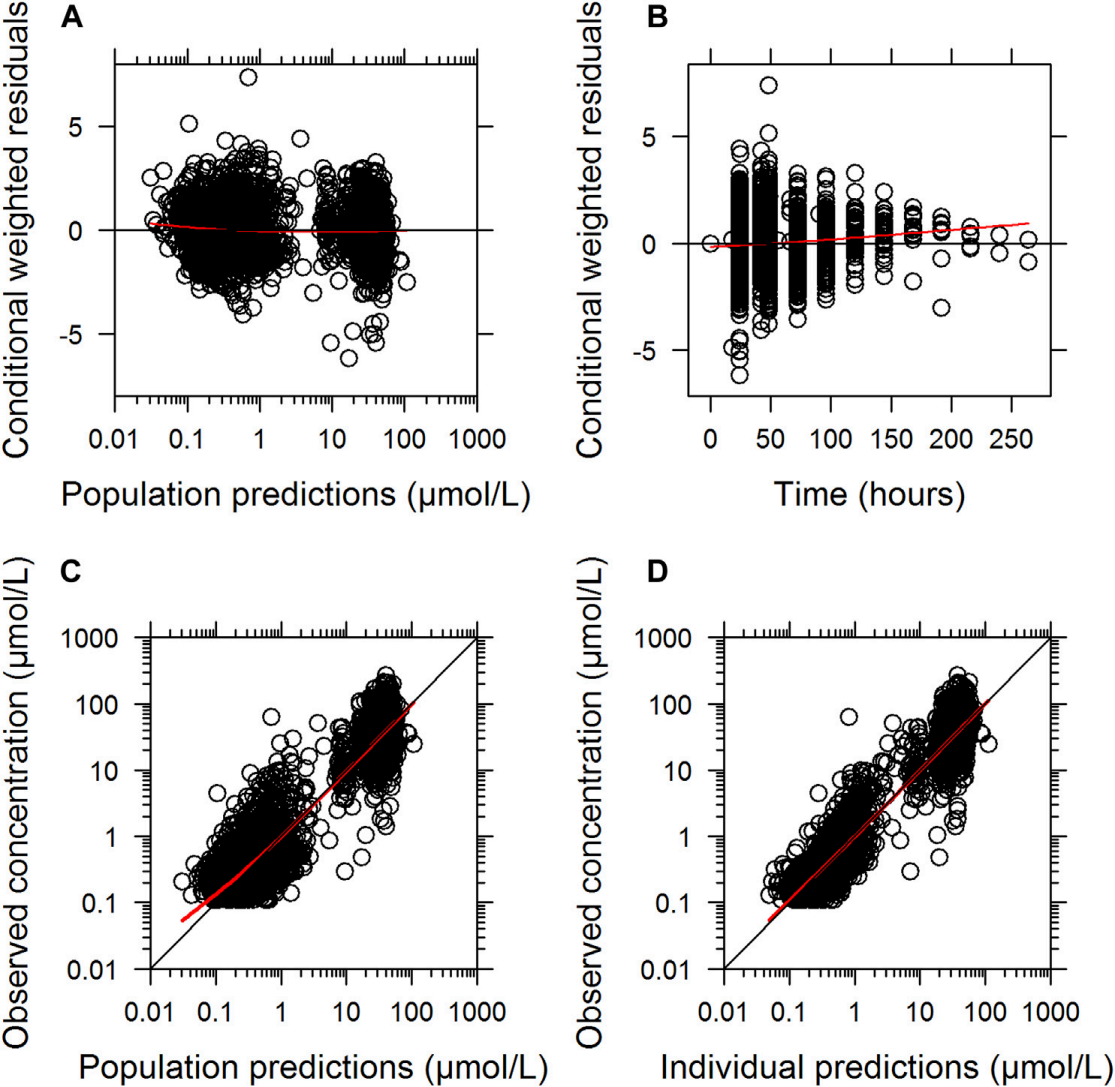
Goodness-of-fit of the final population pharmacokinetic model describing MTX. Conditionally weighted residuals *vs.* population predicted concentrations **(A)**; conditionally weighted residuals *vs.* time **(B)**. Observed plasma concentrations *vs.* population predicted concentrations **(C)**; Observed plasma concentrations *vs.* individually predicted concentrations **(D)**; Solid red lines represent locally weighted least squares regressions.

**FIGURE 2:**
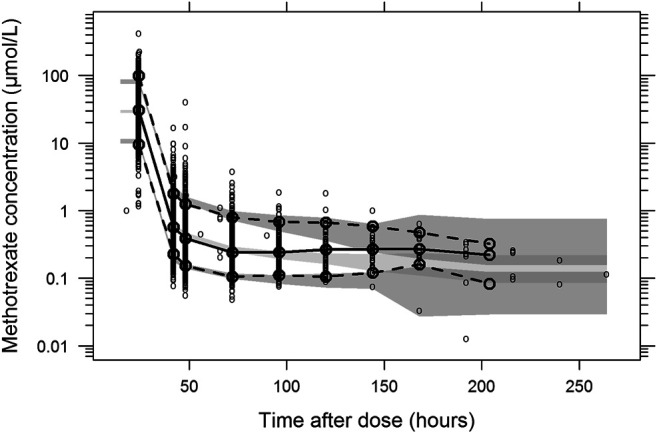
Prediction- and variability-corrected visual predictive check of the final population pharmacokinetic model for methotrexate. Based on 1,000 stochastic simulations. Open circles represent the observations, and solid lines represent the 5th, 50th, and 95th percentiles of the observed data. The shaded areas represent the 95% confidence intervals around the simulated 5th, 50th, and 95th percentiles.

The final parameter estimates showed good precision with relatively small standard errors (<15%), confirming the stability of the model (Table 2). The final parameter estimates described the expected distribution and elimination processes, as well as the associated unexplained variability in the study population. Goodness-of-fit diagnostic plots (Figure 1) and visual predictive checks (Figure 2) demonstrated a good description of the observed data and adequate predictive performance of the final model. Only 7 (0.15%) predictions lied outside the 5 to −5 CWRES for both the population predictions and time graphs. The R^2^ values for OBS vs. PRED and IPRED were 0.882 and 0.925, respectively.

**TABLE 2:** **|** Final population PK parameter estimates of methotrexate in children with ALL.

Parameters	NONMEM estimates	SIR median (95%CI)	CV for IIV	SIR median (95%CI)
CL (L/h)	6.9 (2.5)	6.9 (6.62–7.19)	17.5 (5.8)	17.6 (16.0–19.7)
V_C_ (L)	20.7 (4.9)	20.5 (18.5–22.4)	—	—
Q_1_ (L/h)	0.255 (7.4)	0.258 (0.232–0.285)	26.2 (17.2)	26.2 (18.4–33.6)
V_P1_ (L)	41.0 (11.4)	42.1 (34.4–51.3)	—	—
Q_2_ (L/h)	0.217 (8.7)	0.224 (0.193–0.260)	—	—
V_P2_ (L)	3.17 (9.8)	3.28 (2.75–3.88)	—	—
SCr on CL (%)	−0.97 (4.7)	−0.96 (−0.91 to −1.00)	—	—
σ	0.354 (4.3)	0.350 (0.336–0.366)	—	—

CL is elimination clearance. V_C_ is the central volume of distribution. Q is the inter-compartmental clearance. V_P_ is the peripheral volume of distribution. σ is the additive residue error on the log scale.

Population estimates in Table 2 are given for a “typical” child with body weight of 19 kg. Body weight, was implemented as a fixed allometric function on all clearance and volume of distribution parameters using exponent of 0.75 and 1.0, respectively.

The coefficients of variation for inter-individual variability (IIV) were calculated as 100 × (e^variance^)^1/2^. The relative standard errors (%RSE) were calculated as 100 × (standard deviation/mean).

The SCr was implemented on CL as a linear function [CL = CL_typical_ × ((SCr-26) × 0.0097)].

SIR: Sampling importance resampling approach. The uncertainty was derived from the SIR, with options of 2,000 samples and 1,000 resamples.

## Discussion

The dataset used herein is one of the largest pediatric ALL datasets for Chinese patients receiving HD-MTX chemotherapy, has a good depth of data collected at or beyond 96 h after the start of MTX infusion, and is suitable for determining whether there is an association between AEs and therapeutic outcomes.

HD-MTX remains an important chemotherapy regime for the treatment of patients with ALL. HD-MTX with leucovorin rescue regimen aims to increase MTX concentrations in specific pharmacological sanctuaries (e.g., CNS and testes), results in subsequently increase in cellular MTX uptake in resistant tumor cells, and to overcome intracellular resistance mechanisms (Ackland and Schilsky, 1987). It is a well-established fact that efficacy and toxicity are highly related to drug exposure in a list of chemotherapies. Seidel et al. (1997) found that higher MTX clearance was associated with poorer treatment outcomes in patients with childhood ALL. MTX mainly underwent renal elimination and displayed a high IIV in PK exposure (Evans et al., 1986; Evans et al., 1997; Evans et al., 1998; Schmiegelow, 2009). The population PK model allows the determination of the PK parameters as well as identification of the source of variability. The previous population PK models suggested that a list of covariates [e.g., age (Aumente et al., 2006; Thompson et al., 2007; Colom et al., 2009), body weight (Aumente et al., 2006; Colom et al., 2009), renal function (Dupuis et al., 2008; Fukuhara et al., 2008; Mao et al., 2014), and polymorphisms of transporters (Kim et al., 2012; Liu et al., 2017)] were related to MTX PK exposure.

Unlike the previously published population PK models using the two-compartment disposition model (Wall et al., 2000; Aumente et al., 2006; Faltaos et al., 2006; Piard et al., 2007; Min et al., 2009; Buitenkamp et al., 2010; Jönsson et al., 2011; Watanabe et al., 2014; Beechinor et al., 2019), the current study based on 311 pediatric patients enabled to fit the MTX concentration-time data by using a three-compartment disposition model. The final model demonstrated that the MTX clearance estimate in a typical child with a body weight of 19 kg was 6.9 L/h. This value was similar to those reported previously: 8.8 L/h in 79 ALL pediatric patients with an average body weight of 25.3 kg by Piard et al. (Piard et al., 2007), 7.87 L/h in 64 ALL pediatric patients with an average body weight of 25 kg (Faganel et al., 2011), and 7.73 L/h in 36 ALL pediatric patients with an average body weight of 23.4 kg (Hui et al., 2019). The central distribution of volume estimate in current study was 20.7 L, which was similar to that reported by a previous study (16.7 L) involving 64 pediatric patients with ALL/ML (Faganel et al., 2011) and somewhat higher than that reported by another pediatric study (9.3 L for a typical child with body weight of 20 kg) (Aumente et al., 2006).

Previous MTX population PK studies showed that body weight was more frequently included in the model, even though BSA has been reported as a significant covariate in some studies (Ruhs et al., 2012). Aumente et al. (2006) showed that the body weight was proportional to the MTX clearance with the slope estimate of 0.15, in pediatric patients aged more than 10 years, while in an over proportional manner using an allometric power function with an exponent estimate of 0.876 in those aged less than 10 years. Colom et al. (2009) indicated that the clearance was proportional to the body weight, with 0.55 L/h increase per 1 kg increase in body weight. In current study, body weight was allometrically implemented on all clearance and volume parameters using a fixed exponent of 0.75 and 1.0, respectively, as proposed previously (Holford et al., 2013). This could have significantly improved the model fit.

Since MTX was mainly eliminated through the kidneys, renal impairment could affect the elimination and increase the systemic PK exposure of MTX sequentially. Previous population PK studies on MTX chemotherapy in patients with cancer have reported significant effects of creatinine clearance (CRCL) or SCr on MTX clearance (Fukuhara et al., 2008; Ruhs et al., 2012; Mao et al., 2014). A study on pediatric lymphoblastic malignancies indicated that plasma MTX concentration at 48 h was positively correlated with 24 and 48 h SCr levels, but negatively correlated with 24 and 48 h CRCL (Mao et al., 2014). By using a power function in population PK modeling analysis, Fukuhara et al. found that CRCL was positively correlated with MTX clearance (Fukuhara et al., 2008), with the exponent estimate of 0.112. In current study, SCr was identified as a statistically significant covariate on clearance of MTX, which corresponded with above studies. Moreover, a recent population PK model utilizing approximately 32,000 MTX concentration data from a large-scale pediatric patient population receiving MTX chemotherapy (Taylor et al., 2020) found a non-linear relationship between time-varying SCr and clearance. In the current study, we were unable to find a non-linear relationship between time-varying SCr and clearance; this might be due to the narrow distribution of SCr and the small sample size compared to those of the previous study. Moreover, we predicted the effect of SCr on clearance based on the SCr distribution in our study using the equation used by Taylor et al. The predicted SCr effect on clearance was around ±20%, which was consistent with findings from current modeling analysis.

A few reports indicated that concomitant medication, such as penicillin and NSAIDs, could decrease MTX clearance by 61% and 16%, respectively (Kim et al., 2012). It has been reported that omeprazole could decrease MTX clearance by 27% (Joerger et al., 2006). In the current study, the inclusion of omeprazole and NSAIDs concomitant medication resulted in a statistically significant improvement in model fit. However, the effect of these drugs on MTX PK parameters was not retained in the final model due to a minimal reduction in either the inter-individual or residual variability.

This study had some limitations. 1) The MTX concentration data were obtained retrospectively from a single center, while the data from a multi-center clinical study would be considered to improve the accuracy of population PK modeling analysis. 2) Polymorphisms of transporters (e.g., SLC19A1, SLCO1B1, ABCB1, and ABCG2) have been demonstrated to play a role in MTX PK disposition and could improve the model prediction. However, these polymorphisms were not detected in the current retrospective study and need to be investigated in the future.

## Conclusion

In this study, MTX PK profiles were accurately captured using the proposed population PK model. The body weight and SCr were significant covariates on the PK disposition of MTX. The proposed model combined with the maximum *a posteriori* Bayesian approach could estimate individual PK parameters and optimize personalized MTX therapy for pediatric patients with ALL.

## Data Availability

The original contributions presented in the study are included in the article/supplementary material, further inquiries can be directed to the corresponding author.
